# Supplementary material for: ZrO2 Nanoparticles and Poly(diallyldimethylammonium chloride)-Doped Graphene Oxide Aerogel-Coated Stainless-Steel Mesh for the Effective Adsorption of Organophosphorus Pesticides
Source: Foods. 2021 Jul 13;10(7):1616. doi: 10.3390/foods10071616 (PMC8304140; doi:10.3390/foods10071616)
Supplement: Supplementary file 1 [file foods-10-01616-s001.zip › foods-1246949-supplementary.pdf]

Supplementary Material

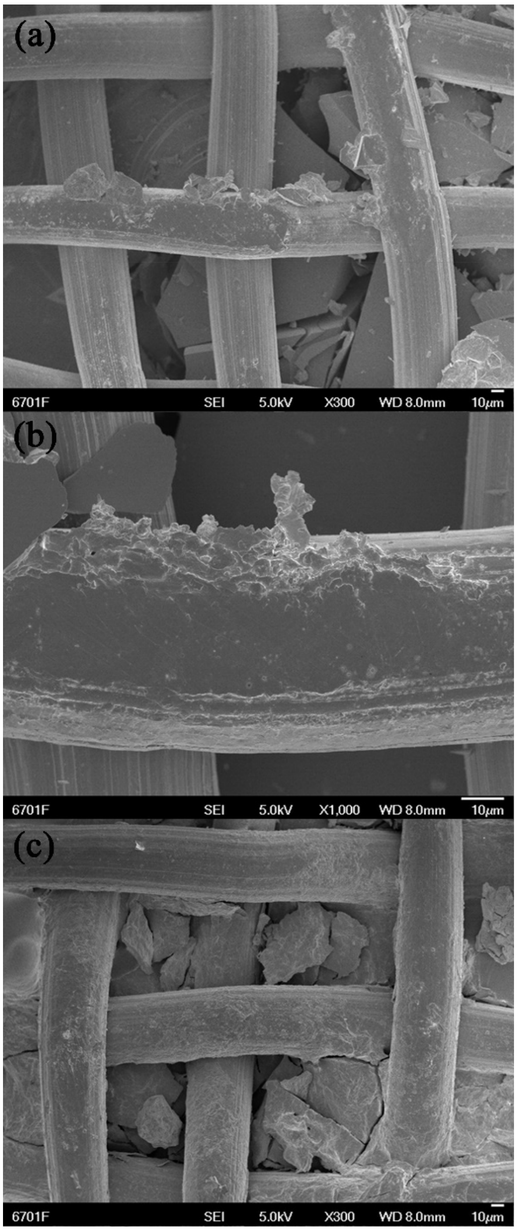

Figure S1. SEM images of the etched SSM, PDDA-GOA-coated SSM, and ZrO_2/PDDA-GOA-coated SSM.

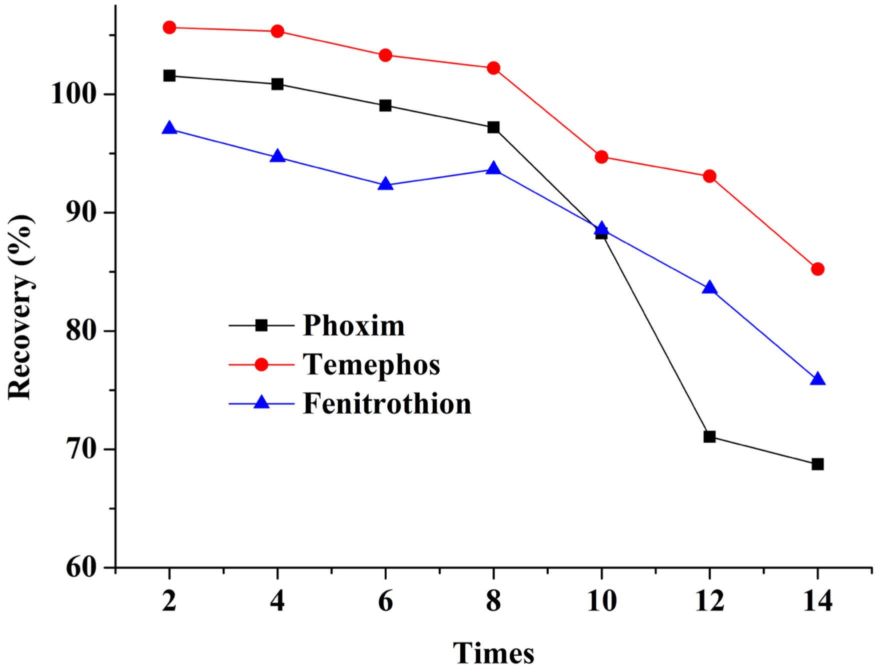

Figure S2. Evaluation of sorbent lifetime after using several times.

Table S1 Comparison of methods used for the determination of OPPs.

Method	Sorbent/extractant	Sample	Sorbent	EF	Linearity (μg L^{-1}, μg kg^{-1})	LOD (μg L^{-1}, μg kg^{-1})	Lifetime (times)	Ref.
MSPE[a]-HPLC-UV	Poly(ionic liquid)/Fe_3O_4	Tea drink	60 mg	84-161	1-200 μg L^{-1}	0.01 μg L^{-1}	20	[1]
HF-SPME[b]-HPLC-UV	MIL-101@GO	Tomato, cucumber and agricultural water	--	41-49	1-500 μg L^{-1}	0.21-0.27 μg L^{-1}	--	[2]
SPE-HPLC-UV	MIP[c]	Water	40 mg	330	50-1000 μg L^{-1}	0.07-0.12 μg L^{-1}	> 50	[3]
MSPE-HPLC-UV	$NiFe_2O_4$@SiO_2@polyaniline -ionic liquid	Fruit juice	15 mg	--	0.21-500 μg L^{-1}	0.06-0.17 μg L^{-1}	8	[4]
QuEChERS[d]-GC/MS	Multi-walled carbon nanotubes	Peanut oil	100 mg	--	5-200 μg kg^{-1}	0.7-1.6 μg kg^{-1}	--	[5]
MSDE[e]-GC/MS	C18	Bovine liver samples	500 mg	--	500-1500 μg kg^{-1}	25-100 μg kg^{-1}	--	[6]
HS-SPME-GC/MS	Polydimethylsiloxane/divinyl benzene	Milk, cows	--	--	14.6-32 μg L^{-1}	2.16-10.85 μg L^{-1}	--	[7]
DSPE[f]-GC/FID	Zinc-based metal organic framework	Water, fruit juice	8 mg	801-914	0.1-100 μg L^{-1}	0.03-0.21 μg L^{-1}	8	[8]
SPE-HPLC/UV	ZrO_2/PDDA-GOA/SSM	Vegetables	--	18.7-20.9	1-200 μg L^{-1}	0.2 μg L^{-1}	8	This study

[a]MSPE, magnetic solid-phase extraction; [b]HF-SPME, hollow fiber solid-phase microextraction; [c]MIP, molecularly imprinted polymer; [d]QuEChERS, Quick, Easy, Cheap, Effective, Rugged, Safe. [e]MSDE, matrix solid-phase dispersion; [f]DSPE, dispersive solid-phase extraction.

References

[1] X. Zheng, L. He, Y. Duan, X. Jiang, G. Xiang, W. Zhao, S. Zhang, Poly(ionic liquid) immobilized magnetic nanoparticles as new adsorbent for extraction and enrichment of organophosphorus pesticides from tea drinks, J. Chromatogr. A 1358 (2014) 39-45.

[2] F. Darvishnejad, J.B. Raoof, M. Ghani, MIL-101 (Cr)@graphene oxide-reinforced hollow fiber solid-phase microextraction coupled with high-performance liquid chromatography to determine diazinon and chlorpyrifos in tomato, cucumber and agricultural water, Anal. Chim. Acta 1140 (2020) 99-110.

[3] P.G. Arias, H.M. Martínez-Pérez-Cejuela, A. Combès, V. Pichon, E. Pereira, J.M. Herrero-Martínez, M. Bravo, Selective solid-phase extraction of organophosphorus pesticides and their oxon-derivatives from water samples using molecularly imprinted polymer followed by high-performance liquid chromatography with UV detection, J. Chromatogr. A 1626 (2020) 461346.

[4] D.C. Chen, S. Ma, X.F. Zhang, X.D. Wang, M. Gao, J.Y. Li, H.L. Wang, Enhanced extraction of organophosphorus pesticides from fruit juices using magnetic effervescent tablets composed of the $NiFe_2O_4$@SiO_2@PANI-IL nanocomposites, RSC Adv. 11 (2021) 1668-1678.

[5] R. Su, X. Xu, X. Wang, D. Li, X. Li, H. Zhang, A. Yu, Determination of organophosphorus pesticides in peanut oil by dispersive solid phase extraction gas chromatography-mass spectrometry, J. Chromatogr. B 879 (2011) 3423-3428.

[6] M.P. García de Llasera, M.L. Reyes-Reyes, A validated matrix solid-phase dispersion method for the extraction of organophosphorus pesticides from bovine samples, Food Chem. 114 (2009) 1510-1516.

[7] F.d.M. Rodrigues, P.R.R. Mesquita, L.S. de Oliveira, F.S. de Oliveira, A. Menezes Filho, P.A. de P. Pereira, J.B. de Andrade, Development of a headspace solid-phase microextraction/gas chromatography–mass spectrometry method for determination of organophosphorus pesticide residues in cow milk, Microchem. J. 98 (2011) 56-61.

[8] A. Amiri, R. Tayebee, A. Abdar, F. Narenji Sani, Synthesis of a zinc-based metal-organic framework with histamine as an organic linker for the dispersive solid-phase extraction of organophosphorus pesticides in water and fruit juice samples, J. Chromatogr. A 1597 (2019) 39-45.
